# The big causes of death from noncommunicable disease

**DOI:** 10.2471/BLT.16.030616

**Published:** 2016-06-01

## Abstract

Richard Peto tells Andréia Azevedo Soares why efforts to reduce premature death from noncommunicable diseases should focus mainly on the big causes.

Q: You wanted to be an astronomer when you were at school. How did you become interested in medical statistics?

A: By accident. At university I stumbled into statistics without really knowing what it was, then in 1967 I had a job interview with Richard Doll (1912–2005), who had been one of the first to show that smoking caused lung cancer. Towards the end of the interview he asked me why I wanted to work with him as a statistician, and I said: “I don’t know if I do want to – in fact, I don’t even know whether I want a job at all.” I remember his wife saying to me at the first Christmas party: “So you’re the young man who isn’t sure whether he wants to work or not. Have you made up your mind yet?” and I said I hadn’t. At that time I still wasn’t sure that going to work every day was the best thing to do on this planet.

Q: Why did you change your mind?

A: A few months later I started to get my first scientific results. It doesn’t matter how important they actually were, but it is really exciting and interesting to get new results. From then on I worked more and more. In those first few months I would never take work home with me. Now, I never go home without a briefcase, but I was probably a better human being when I was young.

Q: What kind of research were you doing?

A: I was working on many different studies, but two were of particular relevance to tobacco. Charles Fletcher (1911–1995) had written the 1962 report of the Royal College of Physicians on the hazards of smoking, which was the first report on smoking by such a major body, and led directly to the highly influential 1964 report of the US Surgeon General on smoking and health. I worked with Fletcher on chronic obstructive pulmonary disease, showing that some people who smoked cigarettes didn’t get much lung damage, but some suffered progressive loss of lung function over many years, leading eventually to disability and death. If, however, those getting into trouble stopped smoking before their health was severely affected, deterioration slowed substantially.

Q: What was the other study?

A: I was also working with Richard Doll on the 20-year follow-up of his study of smoking and death in British doctors. In 1951 Doll had asked all the doctors in Britain whether they themselves smoked, and was following them up to compare the death rates in smokers, ex-smokers and never-smokers. When British doctors read Doll’s findings they realized smoking was *really* serious: it wasn’t just killing patients, it was killing doctors too! So, most of the British medical profession accepted the evidence of hazard, and British doctors became the first major group of serious smokers with widespread permanent smoking cessation, providing a nice natural demonstration that stopping smoking saves lives. Later, cessation spread to other professionals, then to the country as a whole. Doll and I published the 20-year results in the 1970s, the 40-year results in the 1990s and the 50-year results in the 2000s.

Q: Why the focus on smoking rather than other possible causes of cancer?

A: In the 1970s some people, believing that most cancers in non-smokers were due to occupational and environmental pollutants, hoped to use laboratory tests on animals to find out which industrial chemicals were carcinogenic, ban those chemicals, and thereby greatly reduce human cancer death rates. Doll and I thought this was unrealistic, and that the big causes of cancer were probably not industry-derived environmental pollutants. If so, a strategy of over-reliance on animal tests risked neglecting the few really important human hazards, like cigarette smoking. Our focus on human evidence and on the few causes of cancer that were known to be big was regarded by some as old-fashioned, even though in the United Kingdom in the 1970s cigarettes were a cause of well over half of all cancer deaths in men and an increasing proportion of all cancer deaths in women.

Q: How can we know what the big causes are unless we study everything, including environmental contaminants?

A: Many causes of cancer have been discovered in the past few decades, and others await discovery. For example, the risk of specific types of cancer is increased by certain chronic infections, by particular types of mould on poorly stored food, by traditional medicinal herbs containing aristolochic acid, by some hormonal medicines, and by obesity and diabetes. It’s somewhat reassuring, however, that in recent decades non-smoker cancer (and other) death rates in middle age have been decreasing in many countries, so I don’t expect any new causes of noncommunicable disease to be as big as smoking, which still causes about a quarter of all cancer deaths in developed countries. Of course, it’s better if researchers don’t all follow the same ideas, as we might then all be wrong. For me, however, what I wanted in the 1970s – and still want now – is for my work to be on the big causes of premature death in many different populations. A moderate reduction in a big cause can prevent far more deaths than a big reduction in a small cause. Smoking kills and stopping works, but a billion people still smoke.

“A moderate reduction in a big cause can prevent far more deaths than a big reduction in a small cause. Smoking kills and stopping works, but a billion people still smoke.”

Q: What was it like to do research in those days?

A: At first we could do only medium-sized studies, but now we’ve got big studies in many different countries of major causes of chronic disease, including not only smoking but alcohol, adiposity (overweight and obesity), diabetes, high blood pressure and high blood lipids, which mainly affect vascular disease rather than cancer. Some thought that studies of such old risk factors would not find anything new, but there were always new things to learn – smoking predominated in Britain, alcohol in the Russian Federation, adiposity and diabetes in Mexico, and in the present century the benefits of stopping smoking are even bigger than early studies had suggested.

Q: You conducted big epidemiological studies in China. How did you become interested in the Chinese tobacco epidemic?

A: As a statistician, I knew that 99% of the world is not British, so I wanted to know more about the causes of premature death in populous countries. I was lucky to get the opportunity to work with Chinese scientists in the 1980s, and I’ve done so ever since. I was expecting communicable disease to predominate as a cause of death, but our collaborative surveys confirmed that it no longer did so. The surveys also showed a vast increase in cigarette use by men, an increase that would take several decades to have its main effects on mortality. The Chinese tobacco epidemic was still at an early stage in the 1980s, and few were taking it seriously. I suggested setting up systems to monitor the extent to which cigarettes were killing the Chinese people and to monitor the changes in this over decades, and the health minister at the time facilitated this.

Q: Did you see any effect of your research in China on cigarette sales?

A: That’s difficult to say. We did a succession of nationwide studies in the 1980s, the 1990s and the 2000s involving a total of two million people, documenting how the epidemic of tobacco deaths was evolving. In 1990, smoking was causing about 10% of all adult male deaths in China, in the 2010s it’s causing about 20%, and by the 2030s it will be causing about 30%. Our October 2015 *Lancet* paper contains the first reliable description of what is happening to male and female mortality from smoking in China (increasing and decreasing, respectively). I don’t know what the effect of our work was or will be, but at least the Chinese epidemic in recent decades has now been documented, and systems are in place to ensure that it will continue to be documented in future decades.

Q: How does your work relate to treatment of noncommunicable diseases?

A: Vaccination, sanitation and treatment have transformed worldwide mortality rates from communicable disease, and treatment has substantially reduced mortality from injuries and noncommunicable disease, at least in countries with good medical services. Indeed, half my work has involved collaboration with colleagues in Oxford on large international trials and meta-analyses of trials of various treatments for vascular disease and breast cancer.

Q: How well do scientists communicate health risks to lay audiences?

A: Some try to emphasize how much the big risks predominate, but some don’t. If we give the general public a list of a hundred possible causes of cancer, it could divert attention from things like smoking that cause vast numbers of premature deaths. In the European Union, for example, there are 1.3 million deaths every year before age 70. More than a million of these are from noncommunicable diseases, including 0.3 million caused by tobacco. The big causes of premature death from noncommunicable disease are smoking, blood pressure, blood lipids, diabetes and chronic infections, and these few big causes should not be obscured.

Q: What might obscure these big risks?

A: Lots of small or uncertain risks. For example, last year the International Agency for Research on Cancer reported that red meat is “probably carcinogenic”. They didn’t say it’s definitely carcinogenic, only probably, so the lower limit of the range of reasonable uncertainty as to how many cancers it causes is zero. Nevertheless, this report generated widespread media coverage, and probably increased general scepticism about news reports about cancer hazards, including those from tobacco. I’m not saying we should concentrate only on tobacco, but we should not divert too much attention away from the big causes of cancer that are proven and well established.

“Generating reliable evidence and acting on it are both needed, but should often be done by different individuals.”

Q: Should epidemiological researchers develop policies based on their findings to change people’s behaviour?

A: Not necessarily. For scientific results to be trusted, it may be best for those producing them not to be too closely connected with the political process of how those results are used. Generating reliable evidence and acting on it are both needed, but should often be done by different individuals. This is true for both the consequences and the causes of smoking. For example, doubling real prices of a packet of cigarettes reduces consumption by about a third. The World Health Organization and the United Nations have targeted a one-third reduction in smoking by 2030, but the world’s governments earn about US$ 300 billion a year in tobacco taxation and sales. If real cigarette prices stay constant and smoking decreases by a third, then the governments of the world would lose US$ 100 billion a year. But if real prices double because of increased excise taxes, this itself will reduce consumption by a third, and the governments would gain US$ 100 billion a year. This is the scientific evidence, but it’s up to governments and society to decide what to do with it.

